# Pathway-Dependent Effectiveness of Network Algorithms for Gene Prioritization

**DOI:** 10.1371/journal.pone.0130589

**Published:** 2015-06-19

**Authors:** Jung Eun Shim, Sohyun Hwang, Insuk Lee

**Affiliations:** Department of Biotechnology, College of Life Science and Biotechnology, Yonsei University, Seoul, Korea; National Institute of Genomic Medicine, MEXICO

## Abstract

A network-based approach has proven useful for the identification of novel genes associated with complex phenotypes, including human diseases. Because network-based gene prioritization algorithms are based on propagating information of known phenotype-associated genes through networks, the pathway structure of each phenotype might significantly affect the effectiveness of algorithms. We systematically compared two popular network algorithms with distinct mechanisms – *direct neighborhood* which propagates information to only direct network neighbors, and *network diffusion* which diffuses information throughout the entire network – in prioritization of genes for worm and human phenotypes. Previous studies reported that network diffusion generally outperforms direct neighborhood for human diseases. Although prioritization power is generally measured for all ranked genes, only the top candidates are significant for subsequent functional analysis. We found that high prioritizing power of a network algorithm for all genes cannot guarantee successful prioritization of top ranked candidates for a given phenotype. Indeed, the majority of the phenotypes that were more efficiently prioritized by network diffusion showed higher prioritizing power for top candidates by direct neighborhood. We also found that connectivity among pathway genes for each phenotype largely determines which network algorithm is more effective, suggesting that the network algorithm used for each phenotype should be chosen with consideration of pathway gene connectivity.

## Introduction

Genes that are associated with the same phenotypes tend to be co-functional. This functional association between genes can be harnessed to identify novel genes that might be associated with complex phenotypes, for example human diseases [1–3]. Network-based gene prioritization for phenotypes involves four factors: i) gene networks, ii) known genes for a phenotype of interest, iii) algorithms to propagate information of known phenotype genes through the network, and iv) metrics to assess prioritization models.

Over the past several years, many genome-scale gene networks for various organisms, including humans, have become publicly available and have been used for the prediction of novel disease genes [4–7]. Moreover, the number of phenotype annotations for genes has grown rapidly as a result of many new experimental methods such as genome-wide association study, high-throughput gene knockout, and automated phenotype profiling. Although the availability of high-quality gene networks and phenotypic annotations substantially increases prioritizing power, the network algorithms provide further opportunities for improvement.

**Fig 1:**
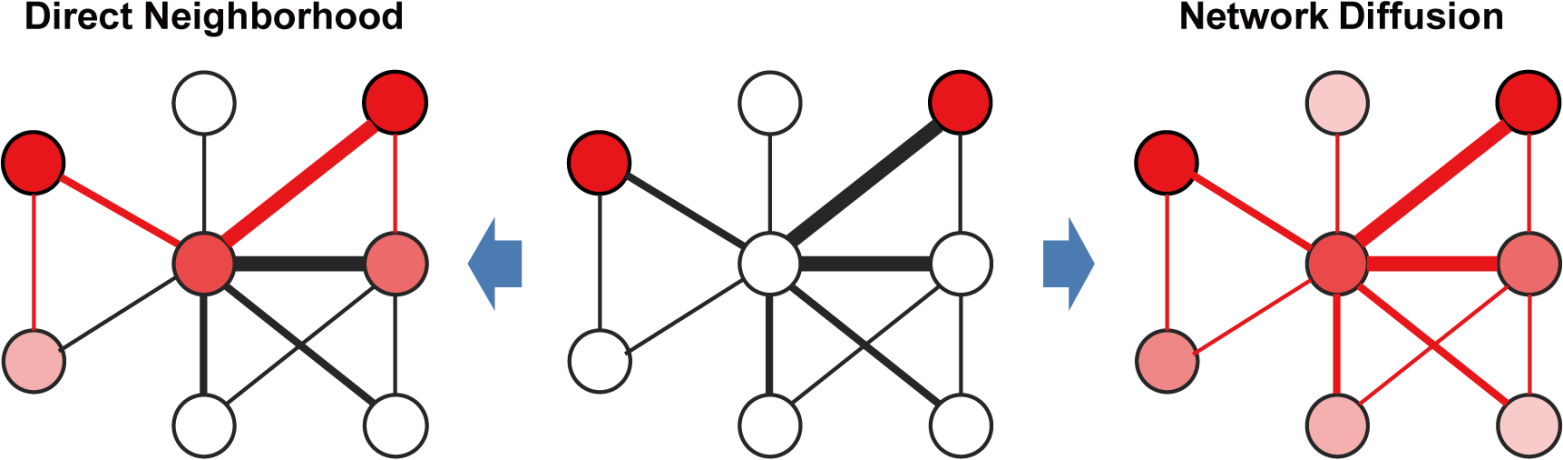
Two categories of network algorithms with distinct mechanisms of propagating information through a network: *direct neighborhood* and *network diffusion*. The score of network edge is indicated by line thickness and that of node information is indicated by the intensity of the red color. Direct neighborhood algorithms propagate node information to direct neighbors only whereas network diffusion algorithms diffuse it throughout the entire network, including indirectly connected neighbors.

Two conceptually distinct algorithms for inference from network neighbors have been widely used [8] (**Fig 1**). In the first, node information can propagate only through direct neighbors, called *direct neighborhood*. In particular, provided a network has edge weight scores, we commonly use a sophisticated type of direct propagation algorithm called naïve Bayes (NB), in which the score of a particular node label is the sum of the network edge weights of all the connected neighbors for the same label. In the second algorithm, node information can diffuse throughout the entire network, called *network diffusion*, such that each node can use information from all other nodes. One network diffusion method that has increased in popularity is Gaussian smoothing (GS) [9]. Conceptually, GS finds solutions where it achieves a minimal difference between the initial and final scores of a labeled gene, and between the label score of a gene and each of its neighbors.

To select the best network algorithm for a given gene prioritization, we also need an adequate measure of prioritization performance. The receiver operating characteristic (ROC) curve, which is summarized as the area under the curve (AUC), is generally used as a measure of model performance. However, the AUC should be interpreted carefully in practical applications for gene prioritization [10] because only the few hundred top candidate genes are generally selected for follow-up functional studies. Therefore, the AUC for ‘early retrieval’ needs to be considered to select the best network algorithm.

In this study, we systematically compared the performance of two distinct network algorithms, NB and GS, in prioritizing all genes or only the top candidate genes for human and worm phenotypes. Our analysis showed that high prioritizing power for all genes does not guarantee successful prioritization for top candidate genes, and that the effectiveness of the two network algorithms for entire ranks and early retrieval are largely affected by pathway gene connectivity in the network. These results provide a set of guidelines for the choice of network algorithms in gene prioritization for a given phenotype.

## Materials and Methods

To test network-based gene prioritization for phenotypes of two animal species, *Caenorhabditis elegans* (worm) and *Homo sapiens* (human), we used WormNet [11] and HumanNet [4], which are genome-scale functional gene networks for *C*. *elegans* and *H*. *sapiens* respectively. For phenotype-associated genes, we used a total of 555 worm RNAi knockdown phenotypes collected from WormBase239 [12] and 761 human disease phenotypes from Disease Ontology (DO) [13]. All phenotypes used in this study contain at least five pathway genes.

The underlying principle of network-based gene prioritization is that genes that lie closer to one another in a gene network are more likely to share functional information. The functional information of a gene can therefore propagate through its network neighbors. We tested two distinct network algorithms, naïve Bayes (NB), which is a direct neighborhood algorithm, and Gaussian smoothing (GS), which is a network diffusion algorithm [8,9] (**Fig 1**). In the NB algorithm, the final score of a particular node label is the sum of the network edge weights of all the connected neighbors for the same label, on the basis of Bayes theorem as following:

$$f_{i}^{final}=P\left(c|X_{i}\right)=P\left(c\right)\times \prod_{j}P\left(X_{j}|c\right)$$

where *P(X|c)* is the probability of node *X* given class label ‘c’, and node *X*_*j*_ represents the neighbors of node *X*_*i*_ in the network. The GS algorithm, propagating labels by Gaussian probability density function, aims to find optimal solution to minimize two differences: i) between the initial and final scores of a labeled node, ii) between the label score of a node and each of its neighbors. Among various implementations of GS algorithm, we used GeneMANIA label propagation algorithm [9]. Unlike many GS algorithms using smoothing parameter for the amount of node information to be diffused throughout network neighbors, GeneMANIA assigns initial bias (as mean label) to the unlabeled nodes, and adopts the harmonic solution that can be computed using matrix methods.

$$f={\text{argmin}}_{\text{f}}\sum_{i}{\left(f_{i}-y_{i}\right)}^{2}+\;\sum_{i}\sum_{j}w_{ij}{\left(f_{i}-f_{j}\right)}^{2}$$

where *y*_*i*_ and *f*_*i*_ indicate the initial and final score of node *X*_*i*_ and *w*_*ij*_ is the edge weight between node *X*_*i*_ and its neighbor *X*_*j*_.

ROC curves are plots of the false positive (FP) rate versus true positive (TP) rate at various thresholds of FP rate. We measured the AUC for the entire threshold range (conventional AUC) as well as the AUC for the top 200 candidate genes (AUC_Top200_) to determine the AUC for early retrieval. Given that only a few hundred genes at most are associated with each phenotype, AUC_Top200_ is a more practical indicator of candidate quality for subsequent functional studies.

## Results

### Emergence of three classes of phenotypes based on patterns of ROC curves using distinct network algorithms

ROC curve analysis is the most widely used method to assess models for classification and prioritization and the result of assessment is commonly summarized by the AUC score. In previous studies, the power of network-based gene prioritization has been evaluated based on AUC. For example, the GS algorithm showed higher performance than NB in prioritizing genes for the majority of human diseases on the basis of the AUC [4,8]. However, high AUC does not necessarily indicate successful prioritization for the top ranked candidates. To address this ‘early retrieval’ problem, several alternative metrics based on mathematically transformed ROC curves have been proposed [14–18]. In this study, to assess the prioritizing power for early retrieved candidates we simply measured AUC scores for only the top N candidates (AUC_TopN_), which requires no further mathematical transformation.

We conducted network-based gene prioritization for phenotypes of human and worm using genome-scale functional gene networks for the two species: HumanNet [4] and WormNet [11]. Prioritization models were assessed for 555 worm RNAi phenotypes derived from WormBase239 [12] and 761 human disease phenotypes from Disease Ontology (DO) [13]. Given that both worm and human have approximately 20,000 coding genes, we decided to measure early retrieval AUC for the top 200 candidates (AUC_Top200_), accounting for approximately 1% of the coding genome for both species.

**Fig 2:**
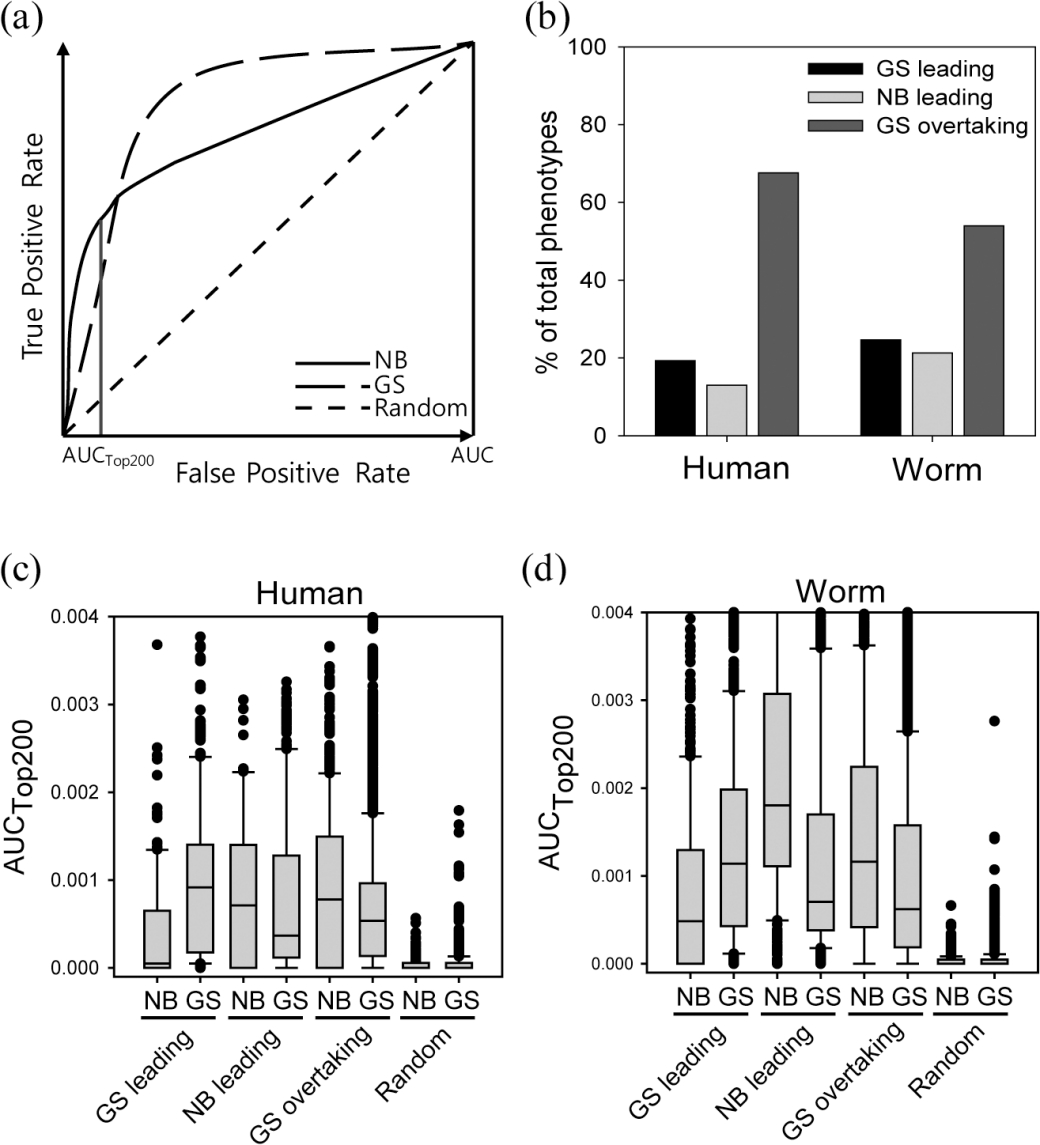
(a) ROC curve pattern for ‘GS overtaking class’. (b) Percentage of 761 human diseases or 555 worm RNAi phenotypes in the three classes as determined by the ROC curve relationship for NB and GS. The ‘GS overtaking class’ is dominant among both human diseases and worm RNAi phenotypes. Distribution of AUC_Top200_ for human diseases (c) and worm RNAi phenotypes (d) measured using disjoint and equal-sized training gene set and test gene sets, simulated by 100 rounds of random splitting of original phenotype gene sets. GS outperforms NB for phenotypes of GS leading class, while NB outperforms GS for phenotypes of NB leading class and GS overtaking class for both human diseases and worm RNAi phenotypes.

To study differences in algorithmic effectiveness for different ranges of gene ranks, we compared ROC curves by NB and GS. Interestingly, worm RNAi phenotypes and human diseases revealed three classes of phenotype sets based on differences in the score of AUC and AUC_Top200_ between NB and GS: i) ‘GS leading class’ where GS consistently outscores NB over the entire range of the ROC curve, ii) ‘NB leading class’ where NB consistently outscores GS over the entire range of the ROC curve, iii) ‘GS overtaking class’ where GS is inferior to NB for AUC for top candidates (e.g., AUC_Top200_) but eventually surpasses NB for entire ranks (**Fig 2A**). We did not define a ‘NB overtaking class’, because the number of phenotypes for which NB significantly overtook GS was negligible.

Consistent with previous observations [4,8], the majority of worm RNAi phenotypes and human diseases were more predictive by network diffusion algorithms (i.e., the sum of the number of phenotypes for the GS leading and GS overtaking classes; **Fig 2B**), suggesting that an algorithm in which information is propagated through indirect network neighbors with diffusion has advantages over one in which information propagates only through direct neighbors in network-based gene prioritization. However, the majority of the phenotypes with higher AUC by GS (e.g., 505 out of 649 human diseases and 276 out of 402 worm RNAi phenotypes) showed higher AUC_Top200_ by NB (i.e., the GS overtaking class). If we consider early retrieval AUC when choosing the network algorithm, GS seems to be no better than, or possibly even worse than, NB for the GS overtaking class of human diseases. These observations indicate that NB could be a better algorithm in prioritizing top candidates for the majority of human diseases and worm RNAi phenotypes. We repeated the same analysis for different rank threshold (e.g., top 100 and top 500, **S1A Fig**) and for other integrated gene networks, Functional Linkage Network (FLN) [19] for human and a STRING [20] for worm (**S1B Fig**), and observed high abundance of the GS overtaking class among phenotypes with higher AUC by GS indicating that the emergence of the three classes of phenotypes was not rank threshold- or network-specific observation. In addition, we found that the observed relationship between NB and GS is not attributed to the specific smoothing parameters of GS algorithms, although the particular algorithm used in this study, GeneMANIA, does not use smoothing parameter (**S2 Fig**).

### Effectiveness of network algorithms differs among the three classes of phenotypes

In the analysis described above, phenotype genes were prioritized in a leave-one-out analysis setting, in which the score of each phenotype gene is determined by the sum of the edge weights to all other phenotype genes. In this test setting, all phenotype genes take turns in a training model and a testing model, therefore training genes and test genes are not completely independent. Moreover, there are many genes for each training model, whereas there is only a single test gene per iteration step, generally resulting in optimistic evaluations. To evaluate prioritization models from a more realistic perspective, we randomly split the gene set into two equally sized subsets for each phenotype, one for ‘training genes’ and the other for ‘test genes’. We then prioritized test genes by propagating information of the training gene through networks using both NB and GS. **Fig 2C and 2D**show the distribution of AUC_Top200_ scores from 100 simulations for human diseases and worm RNAi phenotypes, respectively. As expected, in both human and worm we found that NB is the best performing algorithm for the NB leading phenotypes and GS is the best performing for GS leading phenotypes. We also observed that NB performs better than GS for GS overtaking phenotypes. Given that the majority of human and worm phenotypes belong to the GS overtaking class, this observation suggests that the choice of GS as an optimal algorithm on the basis of AUC may result in a low discovery rate for many phenotypes, and that NB might be a better choice for prioritizing top ranked candidates in general.

### Pathway connectivity underlies the three classes of phenotype by ROC curve patterns

**Fig 3:**
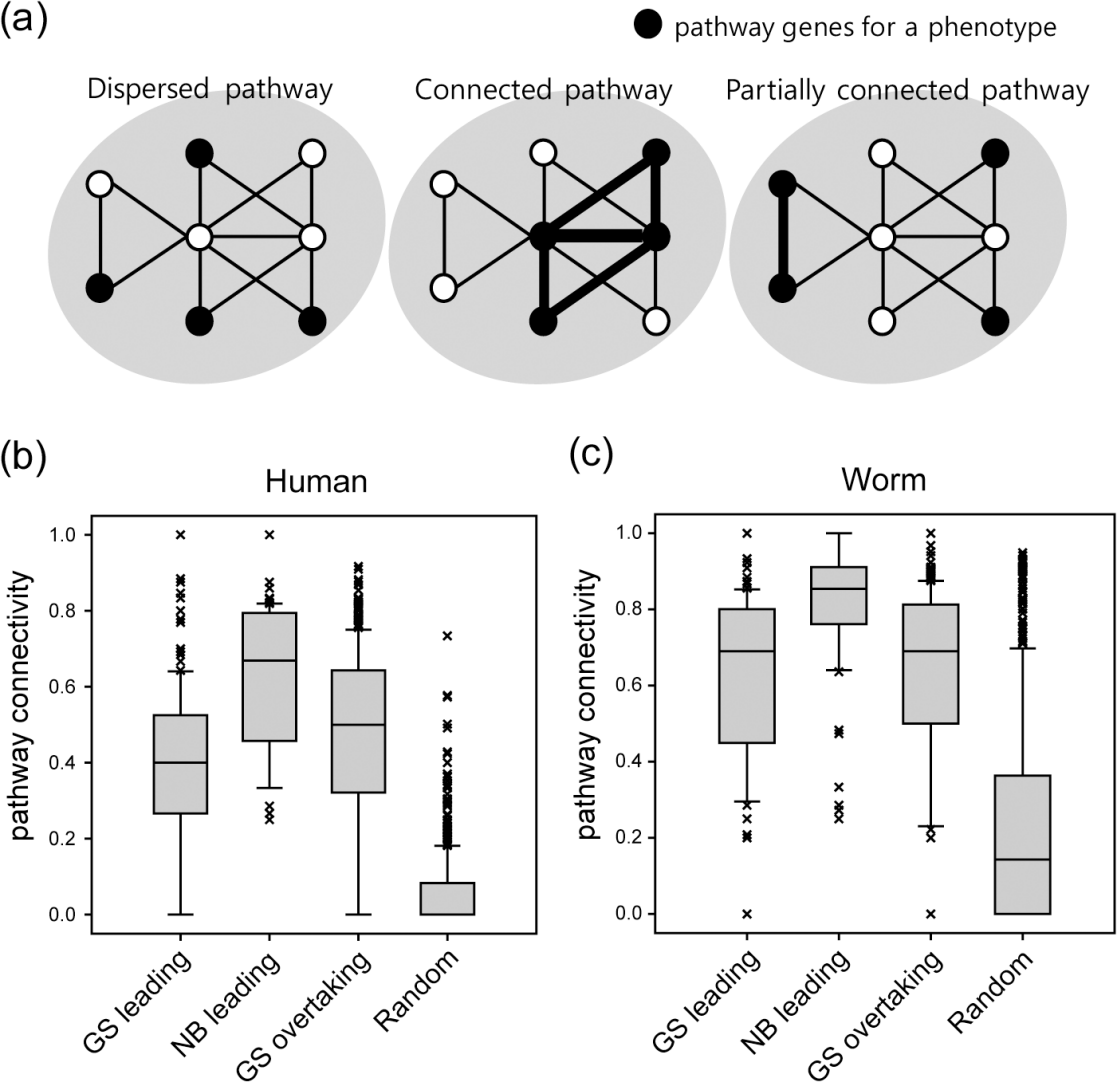
(a) Three hypothetical pathways with different degrees of connectivity. Black nodes represent pathway genes and white nodes represent all other genes of the network. The distributions of pathway connectivity scores of human diseases (b) and worm RNAi phenotypes (c) for each of three classes are shown. The order of pathway connectivity among three phenotype classes was clear for both human diseases and worm RNAi phenotypes: NB leading followed by GS overtaking, then GS leading.

Because network algorithms propagate information through network edges, their algorithmic effectiveness may be affected by connectivity among phenotype genes. We therefore investigated whether network connectivity accounts for the three classes of phenotype based on the relationship between the ROC curves for NB and GS. We hypothesized three different states of pathway connectivity corresponding to the three classes of phenotype: i) a dispersed pathway, ii) a connected pathway, and iii) a partially connected pathway (**Fig 3A**). For phenotypes of dispersed pathways, pathway genes are not directly connected in the network. Hence, only network diffusion algorithms, which can propagate information through indirect neighbors, can prioritize all phenotype genes properly, corresponding to the GS leading class. Conversely, for phenotypes of connected pathways, most pathway genes are directly connected in the network. In this case, the pathway genes are effectively retrieved among top candidates by simply using direct network neighbors (e.g., NB), classifying corresponding phenotypes into the NB leading class. However, it is likely that the majority of phenotype pathways are partially connected. Such pathway gene connectivity will show hybrid properties of prioritization between connected pathways and dispersed pathways. Phenotype genes for the connected part of the pathway will be retrieved among the top candidates by NB, whereas ranks for the disconnected genes will be determined more properly by GS, resulting in a GS overtaking class phenotype.

To validate our hypothesis, we measured the degree of pathway gene connectivity for each phenotype using the *pathway connectivity* score defined as:

$$\mathrm{pathway connectivity}\;=\;\frac{\text{the number of connected pathway genes}}{\text{the total number of pathway genes}}$$

A pathway connectivity of zero represents dispersed pathways and a score of one represents fully connected pathways in the network. **Fig 3B and 3C**shows the distribution of *pathway connectivity* scores for the three classes of phenotypes. For both human diseases and worm RNAi phenotypes, the distribution of *pathway connectivity* scores had the highest range in the NB leading phenotypes, followed by the GS overtaking phenotypes, and then the GS leading phenotypes. This observed hierarchy of *pathway connectivity* scores strongly supports our hypothesis that a different degree of pathway connectivity underlies the three different types of relationship between ROC curves obtained by NB and GS.

**Fig 4:**
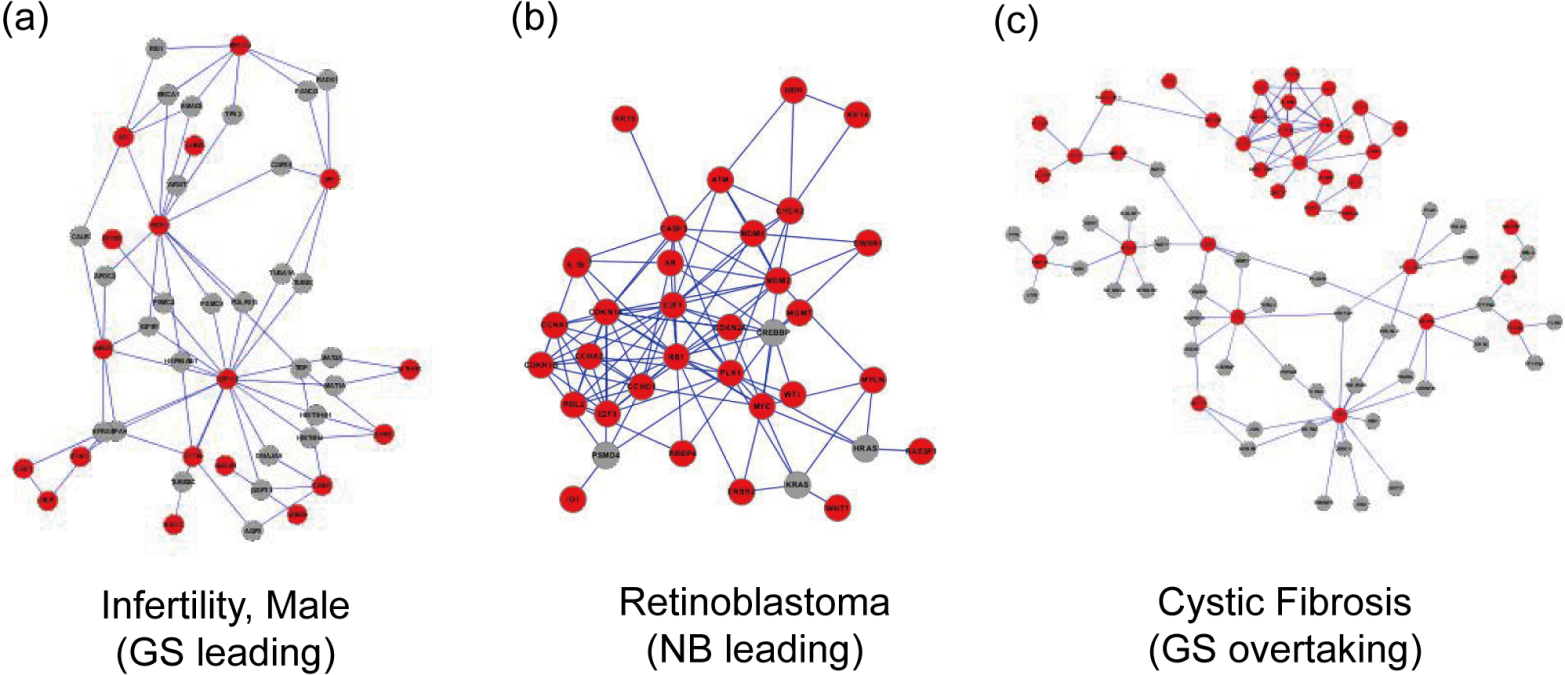
Networks of human disease pathway genes, showing one disease for each of three classes: (a) male infertility for GS leading class, (b) retinoblastoma for NB leading class, and (c) cystic fibrosis for GS overtaking class. Pathway genes for each disease are indicated as red nodes and all others as gray nodes.

Networks of disease pathway genes clearly demonstrate differential degree of connectivity among pathway genes across the three classes of phenotype. In male infertility, a disease of GS leading class, most of the pathway genes are dispersed through the network (**Fig 4A**). Conversely, pathway genes for retinoblastoma, a NB leading disease, are highly connected in the network (**Fig 4B**). For cystic fibrosis, a disease of GS overtaking class, some pathway genes are highly interconnected whereas others are dispersed, accounting for the effective retrieval of pathway genes for top candidates by NB and those for lower ranked candidates by GS (**Fig 4C**).

## Discussion

Network-based gene prioritization is becoming an increasingly popular approach in predictive genetic screens for novel genes associated with pathways and phenotypes in a wide variety of the organisms, from simple microbes to human [2,3]. The core underlying principle of network-based prediction is guilt-by-association (GBA), which assumes that two genes connected in the network operate in the same pathways. The concept of GBA can extend to the prediction of phenotypes, because genes involved in the same phenotype generally belong to the same pathway. The power of network-based gene prioritization is mainly determined by two factors: connectivity among phenotype genes in the network and algorithms to propagate information through the network. In this study, we investigated the contribution of the algorithm to network-based gene prioritization by comparing two distinct network algorithms: NB, which propagates information through only direct neighbors and GS, which propagates information throughout the entire network, including indirectly connected pathway genes, by diffusion. Although the range of propagation of NB is determined by connectivity among pathway genes, that of diffusion algorithms such as GS is much more extensive, generally resulting in significantly higher prioritizing power [8,9].

Algorithmic effectiveness is typically assessed on the basis of AUC, which measures performance of prioritization model for the entire ranks. However, prediction accuracy is more critical among the top ranked genes, which are primarily considered for subsequent functional analysis. Therefore, for both algorithms we measured AUC for the top 200 candidates (AUC_Top200_) in addition to AUC for all genes. Based on the different order for AUC_Top200_ and AUC between NB and GS at two different ranges of ranks, we identified three classes of phenotypes: NB leading (NB wins at both rank ranges), GS leading (GS wins at both rank ranges), GS overtaking (NB wins for top ranks, while GS does for the entire ranks). Unexpectedly, in the majority of the test phenotypes we observed better prioritization for the top candidate genes using the simple NB algorithm, rather than the more advanced GS algorithm. Furthermore, the majority of the phenotypes with higher AUC_Top200_ score by NB show a higher AUC score by GS, thus emerging as the GS overtaking class. This observation suggests that choosing the GS algorithm on the basis of AUC scores could result in suboptimal conditions of network-based gene prioritization.

Taken together, our findings provide practical guidance for performing optimal network-based gene prioritization. First, the choice of network algorithm for gene prioritization should be based on the performance for early retrieval (e.g., AUC_Top200_), because the prioritizing power of network algorithms can differ between results for entire ranks and those for the top ranks, which contain genes more relevant to the original purpose of a predictive genetic screen. Second, the choice of network is more crucial than the choice of search algorithm for successful network-based gene prioritizations. Provided that the given network directly connects most pathway genes for a phenotype (see **Fig 3A**, connected pathway), the simple NB algorithm outperformed the more advanced GS algorithm. GS improves prioritizing power for top candidate genes only if the pathway genes of a phenotype are disconnected from each other in the given network (see **Fig 3A**, dispersed pathway). Last, it might be necessary to use different network algorithms for different phenotypes. For a given network, pathway connectivity varies among phenotypes as a result of differences in either the nature of the pathway structure or the network quality for the given phenotype. Consequently, the effectiveness of a network algorithm would vary accordingly. We observed that phenotypes for three different classes showed optimal performance with different network algorithms, either NB or GS, for different ranges of ranks, and that these classes are related to the properties of pathway connectivity of phenotypes for each class. These data suggest that we should choose whichever algorithm shows best prioritization for each phenotype, rather than using a single algorithm for all phenotypes.

## Supporting Information

S1 Fig Three classes of human diseases or worm RNAi phenotypes. Three classes of human diseases or worm RNAi phenotypes determined by the ROC curve relationship between NB and GS by various threshold of ‘early retrieval’ (AUC_Top100_, AUC_Top200_, and AUC_Top500_ for top 100, 200 and 500 candidates, respectively) with (a) HumanNet and WormNet used in this study or with (b) alternative networks for human (FLN: Functional Linkage Network) and for worm (STRING v9.1).
(TIF)

S2 Fig Effects of GS smoothing parameters on the ROC curve relationship between NB and GS. To study potential effects of smoothing parameters of GS algorithms, we repeated analyses with smoothing parameter of 1%, 10%, 50%, 90%, and 99% for both HumanNet and WormNet. We observed substantially more pathways for GS leasing class and fewer pathways for GS overtaking class by 1% smoothing, indicating that there exists smoothing parameter effects on observed relationship between NB and GS. However, this relationship still observed with 1% smoothing, indicating that the observed ROC curve relationship between NB and GS should be attributed to the smoothing itself rather than specific amount of smoothing.
(TIF)

## References

[1] Ideker T, Sharan R (2008) Protein networks in disease. Genome Res 18: 644–652. 10.1101/gr.071852.107 18381899 PMC3863981

[2] Lee I (2013) Network approaches to the genetic dissection of phenotypes in animals and humans. Anim Cells Syst 17: 75–79.

[3] Lehner B, Lee I (2008) Network-guided genetic screening: building, testing and using gene networks to predict gene function. Brief Funct Genomic Proteomic 7: 217–227. 10.1093/bfgp/eln020 18445637

[4] Lee I, Blom UM, Wang PI, Shim JE, Marcotte EM (2011) Prioritizing candidate disease genes by network-based boosting of genome-wide association data. Genome Res 21: 1109–1121. 10.1101/gr.118992.110 21536720 PMC3129253

[5] Linghu B, Snitkin ES, Hu Z, Xia Y, Delisi C (2009) Genome-wide prioritization of disease genes and identification of disease-disease associations from an integrated human functional linkage network. Genome Biol 10: R91 10.1186/gb-2009-10-9-r91 19728866 PMC2768980

[6] Rhodes DR, Tomlins SA, Varambally S, Mahavisno V, Barrette T, Kalyana-Sundaram S, et al (2005) Probabilistic model of the human protein-protein interaction network. Nat Biotechnol 23: 951–959. 16082366 10.1038/nbt1103

[7] Wu G, Feng X, Stein L (2010) A human functional protein interaction network and its application to cancer data analysis. Genome Biol 11: R53 10.1186/gb-2010-11-5-r53 20482850 PMC2898064

[8] Wang PI, Marcotte EM (2010) It's the machine that matters: Predicting gene function and phenotype from protein networks. Journal of Proteomics 73: 2277–2289. 10.1016/j.jprot.2010.07.005 20637909 PMC2953423

[9] Mostafavi S, Ray D, Warde-Farley D, Grouios C, Morris Q (2008) GeneMANIA: a real-time multiple association network integration algorithm for predicting gene function. Genome Biology 9. 10.1186/gb-2008-9-s1-s4 PMC2447538 18613948

[10] Berrar D, Flach P (2012) Caveats and pitfalls of ROC analysis in clinical microarray research (and how to avoid them). Brief Bioinform 13: 83–97. 10.1093/bib/bbr008 21422066

[11] Lee I, Lehner B, Vavouri T, Shin J, Fraser AG, Marcotte EM (2010) Predicting genetic modifier loci using functional gene networks. Genome Res 20: 1143–1153. 10.1101/gr.102749.109 20538624 PMC2909577

[12] Yook K, Harris TW, Bieri T, Cabunoc A, Chan J, Chen WJ, et al (2012) WormBase 2012: more genomes, more data, new website. Nucleic Acids Res 40: D735–741. 10.1093/nar/gkr954 22067452 PMC3245152

[13] Schriml LM, Arze C, Nadendla S, Chang YW, Mazaitis M, Felix V, et al (2012) Disease Ontology: a backbone for disease semantic integration. Nucleic Acids Res 40: D940–946. 10.1093/nar/gkr972 22080554 PMC3245088

[14] Clark RD, Webster-Clark DJ (2008) Managing bias in ROC curves. J Comput Aided Mol Des 22: 141–146. 10.1007/s10822-008-9181-z 18256892

[15] Sheridan RP, Singh SB, Fluder EM, Kearsley SK (2001) Protocols for bridging the peptide to nonpeptide gap in topological similarity searches. J Chem Inf Comput Sci 41: 1395–1406. 11604041 10.1021/ci0100144

[16] Swamidass SJ, Azencott CA, Daily K, Baldi P (2010) A CROC stronger than ROC: measuring, visualizing and optimizing early retrieval. Bioinformatics 26: 1348–1356. 10.1093/bioinformatics/btq140 20378557 PMC2865862

[17] Truchon JF, Bayly CI (2007) Evaluating virtual screening methods: good and bad metrics for the "early recognition" problem. J Chem Inf Model 47: 488–508. 17288412 10.1021/ci600426e

[18] Zhao W, Hevener KE, White SW, Lee RE, Boyett JM (2009) A statistical framework to evaluate virtual screening. BMC Bioinformatics 10: 225 10.1186/1471-2105-10-225 19619306 PMC2722655

[19] Linghu B, Snitkin ES, Hu ZJ, Xia Y, DeLisi C (2009) Genome-wide prioritization of disease genes and identification of disease-disease associations from an integrated human functional linkage network. Genome Biology 10. 10.1186/gb-2009-10-9-r91 PMC2768980 19728866

[20] Franceschini A, Szklarczyk D, Frankild S, Kuhn M, Simonovic M, Roth A, et al (2013) STRING v9.1: protein-protein interaction networks, with increased coverage and integration. Nucleic Acids Res 41: D808–815. 10.1093/nar/gks1094 23203871 PMC3531103

