# Supplementary figures and images for: Pathway-Dependent Effectiveness of Network Algorithms for Gene Prioritization
Source: PLoS One. 2015 Jun 19;10(6):e0130589. doi: 10.1371/journal.pone.0130589 (PMC4474432; doi:10.1371/journal.pone.0130589)

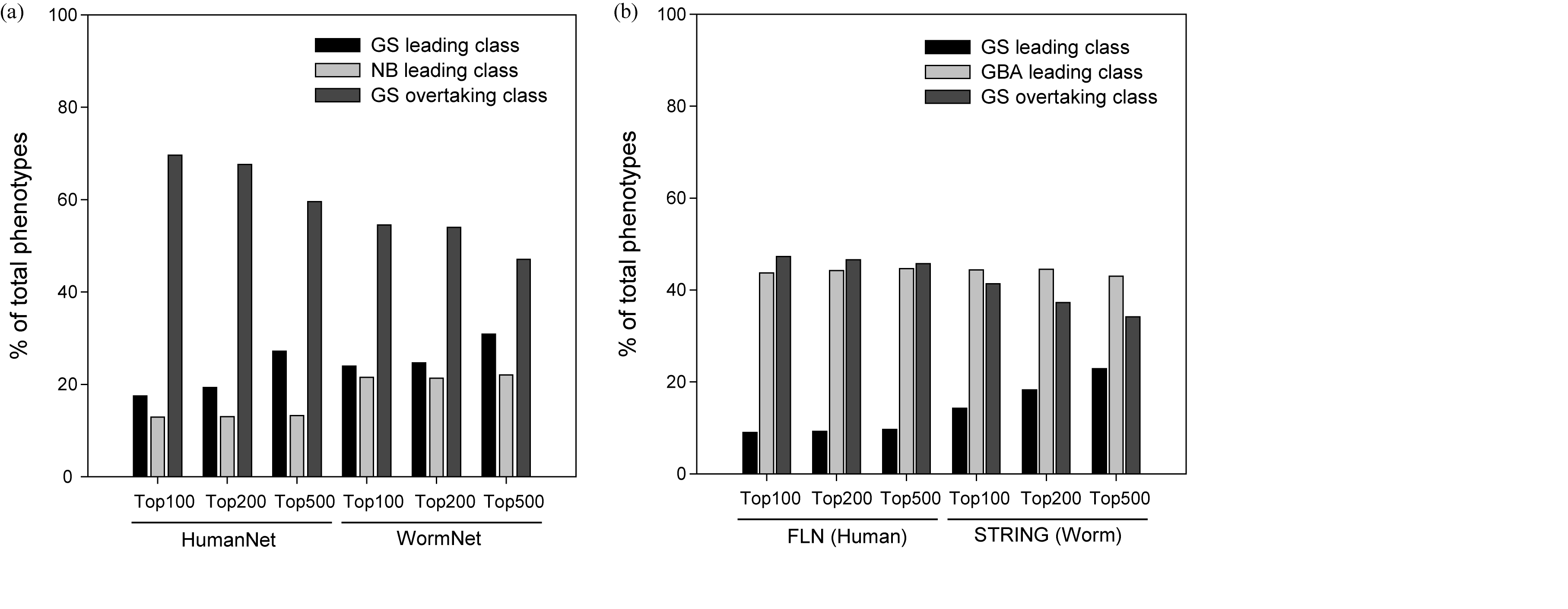

Supplement: S1 Fig — Three classes of human diseases or worm RNAi phenotypes determined by the ROC curve relationship between NB and GS by various threshold of ‘early retrieval’ (AUCTop100, AUCTop200, and AUCTop500 for top 100, 200 and 500 candidates, respectively) with (a) HumanNet and WormNet used in this study or with (b) alternative networks for human (FLN: Functional Linkage Network) and for worm (STRING v9.1). (TIF) [file pone.0130589.s001.tif]

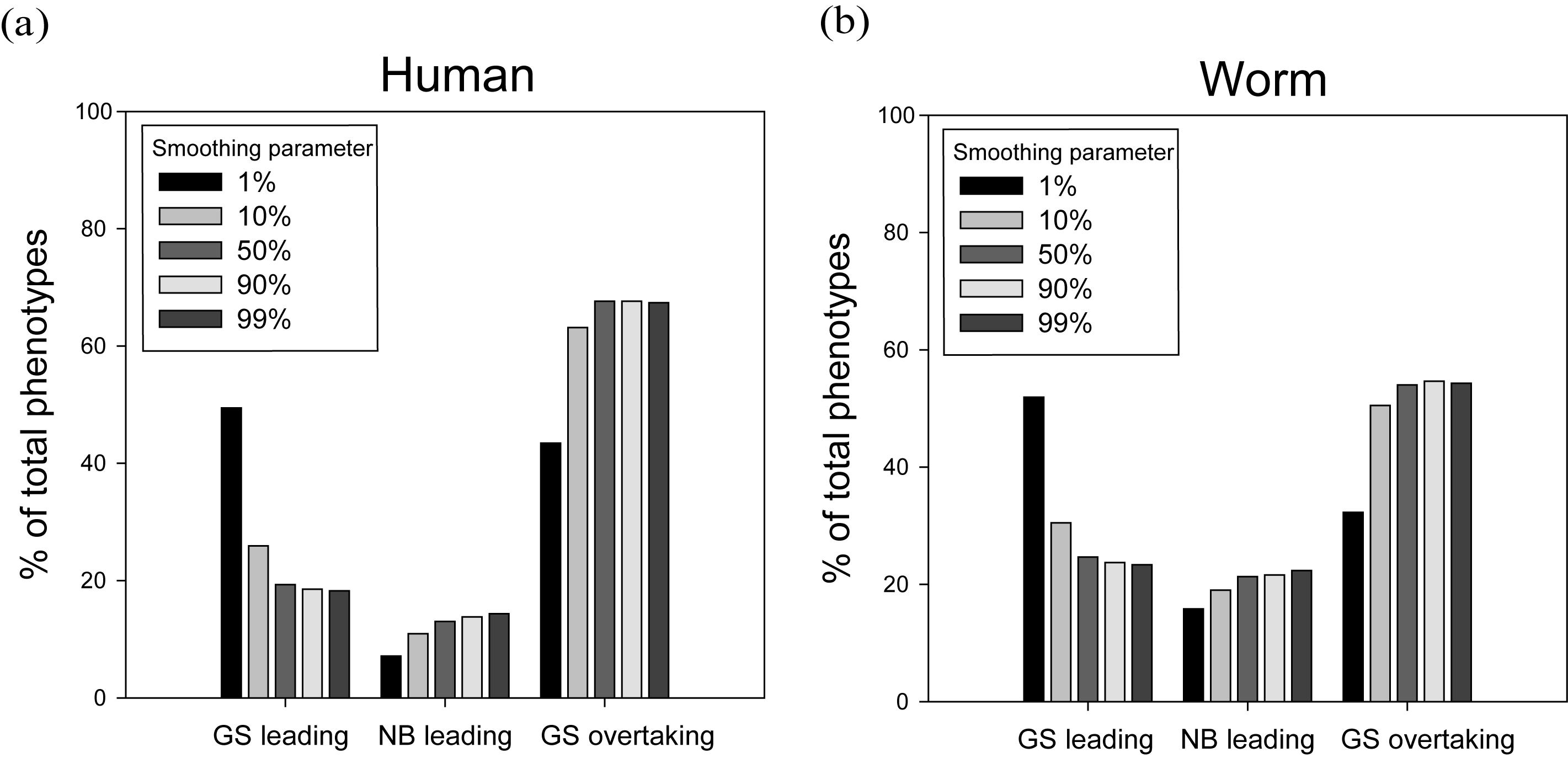

Supplement: S2 Fig — To study potential effects of smoothing parameters of GS algorithms, we repeated analyses with smoothing parameter of 1%, 10%, 50%, 90%, and 99% for both HumanNet and WormNet. We observed substantially more pathways for GS leasing class and fewer pathways for GS overtaking class by 1% smoothing, indicating that there exists smoothing parameter effects on observed relationship between NB and GS. However, this relationship still observed with 1% smoothing, indicating that the observed ROC curve relationship between NB and GS should be attributed to the smoothing itself rather than specific amount of smoothing. (TIF) [file pone.0130589.s002.tif]
